# The structure of winter phytoplankton in Lake Nero, Russia, a hypertrophic lake dominated by Planktothrix-like Cyanobacteria

**DOI:** 10.1186/2046-9063-9-18

**Published:** 2013-09-30

**Authors:** Olga Babanazarova, Sergey Sidelev, Svetlana Schischeleva

**Affiliations:** 1 Yaroslavl State University, Matrosova 9, Yaroslavl 150057, Russia

## Abstract

**Background:**

The permanent dominance of *Planktothrix*-like сyanobacteria has been often reported for shallow eutrophic\hypertrophic lakes in central Europe in summer\autumn. However studies on phytoplankton growth under ice cover in nutrient-rich lakes are very scarce. Lake Nero provides a good example of the contrasting seasonal extremes in environmental conditions. Moreover, the ecosystem underwent a catastrophic transition from eutrophic to hypertrophic 2003–05, with dominance of filamentous cyanobacteria in summer\autumn. Towards the end of the period of ice cover, there is an almost complete lack of light and oxygen but abundance in nutrients, especially ammonium nitrogen, soluble reactive phosphorus and total phosphorus in lake Nero. The aim of the present study was to describe species composition and abundance of the phytoplankton, in relation to the abiotic properties of the habitat to the end of winters 1999–2010. We were interested if *Planktothrix*-like сyanobacteria kept their dominant role under the ice conditions or only survived, and how did the under-ice phytoplankton community differ from year to year.

**Results:**

Samples collected contained 172 algal taxa of sub-generic rank. Abundance of phytoplankton varied widely from very low to the bloom level. Cyanobacteria (*Limnothrix*, *Pseudanabaena*, *Planktothrix*) were present in all winter samples but did not always dominate. Favourable conditions included low winter temperature, thicker ice, almost complete lack of oxygen and high ammonium concentration. Flagellates belonging to Euglenophyta and Cryptophyta dominated in warmer winters, when phosphorus concentrations increased.

**Conclusion:**

A full picture of algal succession in the lake may be obtained only if systematic winter observations are taken into account. Nearly anoxic conditions, severe light deficiency and high concentration of biogenic elements present a highly selective environment for phytoplankton. Hypertrophic water bodies of moderate zone covered by ice in winter and dominated by *Planktothrix* - like сyanobacteria in summer/autumn may follow several scenarios in the end of winter. It may be intense proliferation сyanobacteria normally dominating in summer, or the switch to the other species like the euglenoids and cryptomonads flagellates, or almost total depletion of phytoplankton.

## Background

In temperate lakes, conditions for the development of phytoplankton in winter differ greatly from those in summer. Conventional models of phytoplankton seasonal succession assume a minimum of phytoplankton biomass under ice, due to a combination of unfavorable factors, especially low temperature, short days and poor light penetration - unless the ice is almost transparent. These winter survivors give rise to a new algal community providing the earlier start advantage to the aboriginal species and thereby influencing summer species composition. The degree of under-ice depletion of algal community remains understudied, although some studies of shallow eutrophic lakes in the more temperate climatic zones of Europe and America, emphasized the importance of contrasting conditions under the ice and of the open-water periods for annual algal succession [1-10].

Nevertheless, a few early studies noted mass development of phytoplankton under clear ice. Thus, Yasnitsky’s [11] study in Lake Baikal during 1926–1928 was among the first that reported the development of winter algal blooms. He noted that in 'Melosira years’ [11,12] favorable light conditions under clear spring skies promoted phytoplankton growth below the transparent, firm, snow-free ice. Since that time, proliferation of phytoplankton to bloom levels at the end of the ice-covered period has been repeatedly reported from other lakes [13-19].

This points at the possibility of special importance of the under-ice period in maintaining of a lake’s ecological situation as a whole. Lake Nero (latitude, 57°06’ to 57 °12’ N; longitude, 39 °21’ to 39° 30’ E) provides a good example of the contrasting seasonal extremes, allowing a detailed study of the link between summer algal communities and the under-ice biota. The lake has been intensely studied during the past few decades [20-22]. Lake Nero is a shallow, polymictic, slightly alkaline hypertrophic lake with low transparency and high TN/TP ratio, dominated typically by solitary, filamentous Cyanobacteria, from the beginning of summer to late autumn [20,22]. Following the classical phytosociological nomenclature, Reynolds et al. [23] term the preponderance of this group of filamentous Oscillatoriales as *Planktotrichetum* - S1 codon. Species that make up this assemblage (*Planktothrix agardhii* Gom, *Limnothrix redekei* (Van Goor) Meffert, *Pseudanabaena limnetica* (Lemm.) Kom.), share the trait of dispersion in the plankton as solitary filaments, have superior light interception properties, and a propensity to extreme photoadaptation in turbid, oligophotic conditions. These species maintain a near steady-state within the phytoplankton community of eutrophic/hypertrophic lakes [24]. A sudden shift to an overwhelming dominance of these S1 codon organisms occurred during 2003–2005 in summer/autumn in the lake [20,22]. Here we use the long-term observations of the algal community of Lake Nero to describe the patterns of species composition through which the lake’s ecosystem passes at the end of winter paying special attention to the winter fate of the *Planktotrichetum* codon dominating in summer.

## Results

### Abiotic factors

Lake Nero is covered by ice from November-December to April-May. Thus, the period of ice cover may last for 148–211 days. Annual mean temperature in the Yaroslavl Province grew approximately by 1.2°C during last 50 years. Simultaneously winter temperature increased by 1.7°C. Precipitation increased too. All these changes caused steady water level increase of the Lake Nero observed since 1976. The lake's surface clears from ice now by five days earlier than half a century ago. The dates of freezing did not change [25]. There is a considerable year-to-year variability in ice thickness. The maximum recorded value was 70 cm near Rostov town (Table 1). The structure of the ice cover is typically sponge-like and its transparency is further reduced by a 25–30 cm thick snow layer. The near-bottom temperature is 3.5-4.4 due to high thermo-capacity and intensive reduction processes in sapropel [26]. Total dissolved salts were usually twice as high as in summer due to freezing out (Table 1).

**Table 1:** Abiotic variables in the end of winter

**NH**_**4**_^**+ **^**- N, mg L**^**-1**^	**NO**_**3**_^**—- **^**- N, mg L**^**-1**^	**PO**_**4**_^**3—**^**P, mg L**^**-1**^	**TP, mg L**^**-1**^	**O**_**2, **_**mg L**^**-1**^	**TDS, mg L**^**-1**^	**T, °C**	**Ice, cm**
1.36 ± 0.82	0.34 ± 0.25	0.04 ± 0.02	0.07 ± 0.03	1.43 ± 1.85	420 ± 100	1.75 ± 0.9	51.5 ± 14.8
0.31 – 2.9	0 – 0.8	0.01 – 0.1	0.02 – 0.1	0.0 – 5.3	280 – 540	0.5 – 3.2	25 – 70

Over the line the average value with standard error, under the line minimum and maximum values, n = 32.

Oxygen is rapidly consumed under the ice so that, before the ice finally melts in spring, only trace concentrations of O_2_ remain and the water smells of sulphide. In approximately half of all the samples collected from under the ice, oxygen was not detected at all. In 2003 and 2005, mass fishkill occurred. In the milder winters, openings in the ice cover often appeared. Detectable amounts of methane and CO_2_ are vented through these openings. They may also increase slightly the input of oxygen into the water. In 2008, a large (ca 20 m wide), stable opening developed as a consequence of industrial activity on the shore. Yet, in samples collected only 10 m from the opening, oxygen concentration remained low (1.6 mg L^-1^).

**Figure 1:**
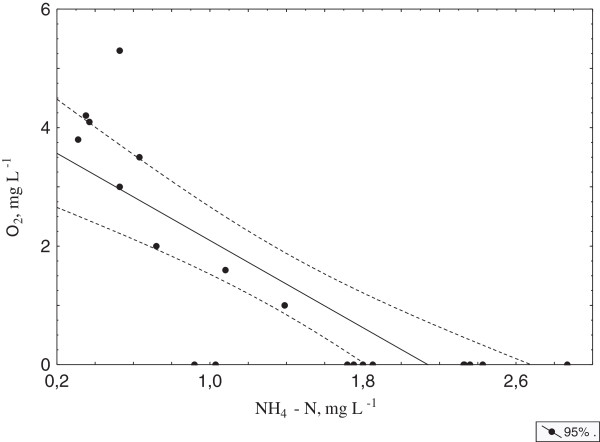
Correlation between ammonium and oxygen concentration in the water under ice.

The under-ice concentrations of other biogenic elements are generally at their highest annual levels at this time [20,25]. Non-parametric correlation analysis of abiotic and biotic parameters revealed a number of significant relationships: ammonium concentrations are inversely correlated to those of nitrate and of oxygen (Table 2). In the absence of oxygen, the concentration of ammonium is extremely high (Figure 1); Spearman correlation between these parameters was - 0.85. Oxygen content was positively correlated with the water level the previous week and negatively correlated with ice thickness.

**Table 2:** Coefficients of nonparametric Spearman correlation

	**NO**_**3**_^**-**^**- N**	**P total**	**Ice**	**O**_**2**_	**Water level**	**В Cyan.%**	**B Flag.%**	**Chl - *a***	**B total**
**NH**_**4**_^**+**^**-N**	-0.8*	0.1	0.5*	-0.9*	-0.4*	-0.1	-0.0	0.1	-0.1
**NO**_**3**_^**-**^**-N**		0.1	-0.4*	0.6*	0.4	-0.1	0.1	-0.2	-0.1
**P total**			-0.3	-0.1	-0.4*	-0.8*	0.7*	0.0	-0.1
**Ice**				-0.7*	0.0	0.4	-0.4	-0.0	-0.3
**O**_**2**_					0.6*	0.1	-0.0	0.1	0.2
**Waterlevel**						0.5*	-0.5*	0.1	-0.1
**В Cyan.%**							-0.8*	0.1	0.1
**B Flag.%**								0.0	0.1
**Chl – *a***									0.7*

Asterisk (*) denotes a significant differences at р<0.05. NH_4_+-N – ammonium nitrogen; NO_3_^—^ -N – nitrate nitrogen; P total – Total phosphorus; Ice – the thick of the ice; O_2_ – oxygen concentrations; Water level – the level of the water; B Cyan.% - the percent Cyanophyta from total biomass; B Flag.% - the percent Euglenophyta and Cryptophyta flagellates from total biomass; Chl – *a* – chlorophill *a* concentrations, B total – Total Biomass.

### Taxonomic composition of the under-ice phytoplankton

Over the full period of our studies, 172 algal taxa of sub-generic rank were encountered in samples collected from under ice in Lake Nero (Additional file 1). The groups were Chlorophyta: 44 taxa, Euglenophyta – 35, Bacillariophyta – 31, Cyanophyta – 27, Chrysophyta – 14, Cryptophyta – 11, Dinophyta – 9 and Xantophyta – 1. The number of species occurring simultaneously in our counts varied from 11 to 55.

The composition of the phytoplankton community under ice generally involved species that are present as vegetative stocks through most of the year. *Limnothrix redekei*, *Pseudanabaena limnetica*, *Planktolyngbya limnetica* may proliferate to dominant proportions in summer as well as in winter. On the other hand, such summer dominants as *Microcystis aeruginosa* (Kütz.) Kütz., *Aphanizomenon gracile* (Lemm.) Lemm., *Scenedesmus communis* Hegewald, fall to low levels in the winter. The only species that was found in all winter samples was *Limnothrix redekei*. *Pseudanabaena limnetica* was present in about 80% of samples. The frequencies of *Planktothrix agardhii*, *Euglena acus* Ehr., *Cryptomonas ovata* Ehr. and *Aulacoseira ambigua* (Grun.) Sim. were each about 70%. Other species were found in less than half of the samples. The species found only in winter samples were: the cyanophyte *Pseudanabaena acicularis* (Nyg.), the dinophyte *Woloszynskia tenuissima* (Lauterb.) Thomp, the chlorophyte *Chlorogonium maximum* Skuja, the cryptophyte С*hroomonas minima* Czosnow and the euglenophyte *E*. *acus* var. *minor* Hansg.

From eco-geographical point of view the under-ice phytoplankton of Lake Nero is a cosmopolitan one with some prevalence of planktonic species indifferent to salinity; there is a significant presence of oligo-*β* and *β*-mesosaprobic forms.

The Sorensen coefficient of compositional similarity between winter algal communities in different years varied from 9.2 to 33.3, indicating significant changes of species compositions in winter.

### Abundance of the phytoplankton

**Figure 2:**
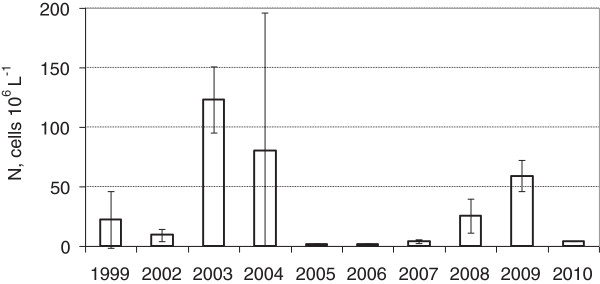
The average total phytoplankton Number with standard deviation.

**Figure 3:**
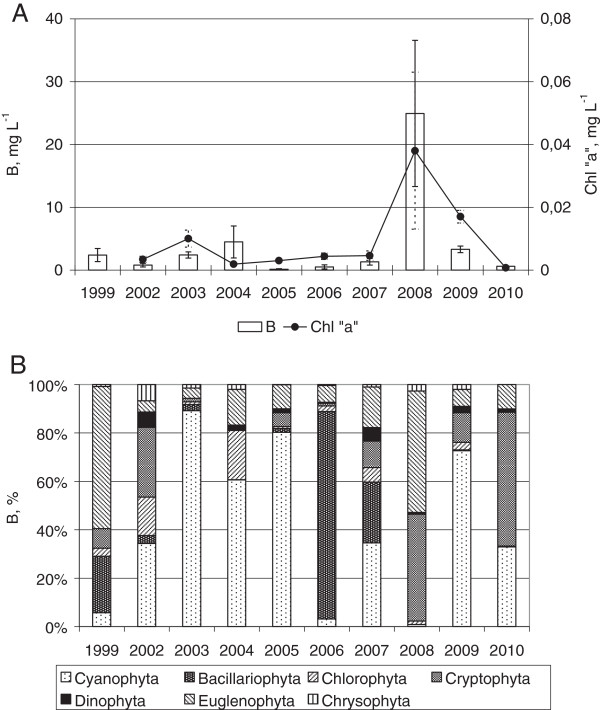
The average of total biomass, chlorophyll "a" concentration with standard deviation (A), percent biomass for all phytoplankton taxa (В).

The total abundance of phytoplankton under the ice and the Shannon index of diversity, as calculated from the biomass values, varied through the winter (Table. 3). Most frequently, the cell concentration and biomass remained relatively low, not higher then 4 million cells L^-1^ or 3 mg L^-1^ (Figures 2 and 3). The maximum biomass was obtained in 2008 at the point N 8 (36.6 mg L^-1^). In most cases the biomass was higher near to the bottom. Average cell volume varied according to the species composition. There were no significant dependencies between total phytoplankton biomass under the ice and in May as well in September. The same holds if only S1 codon biomass is taken into account.

**Table 3:** Biotic parameters

**Chlorophyll *a*, μg L**^**-1**^	**Total phytoplankton Biomass,**	**Total phytoplankton Number,**	**Cell Volume,**	**Shannon index,**
	**mg L**^**-1**^	**10**^**6 **^**cells L**^**-1**^	**micrometr**^**3**^	**bits information/biomass**
10.14 ±14.87	3.65 ± 8.3	31.14 ± 50.52	259 ± 315	2.21 ± 0.86
1.26 – 3.5	0.035 – 36.6	0.0003 – 154.8	20 – 1062	0.5 – 4.0

Top line the average value with standard error, bottom line minimum and maximum values, n = 32.

The concentrations of chlorophyll *a* under ice varied together with the biomass (Figure 3A). The maximal value (63.5 μg L^-1^) was found in March 2008 at the sampling point N 8. Much more commonly, values fell between 1 and 3 μg L^-1^, coinciding with low abundance of phytoplankton in winter. The high concentration of the phaeopigments was a typical feature of phytoplankton collected under the ice, accounting for 40 – 75% of the measured total Chl а. This probably reflects a high proportion of moribund or dead cells.

**Figure 4:**
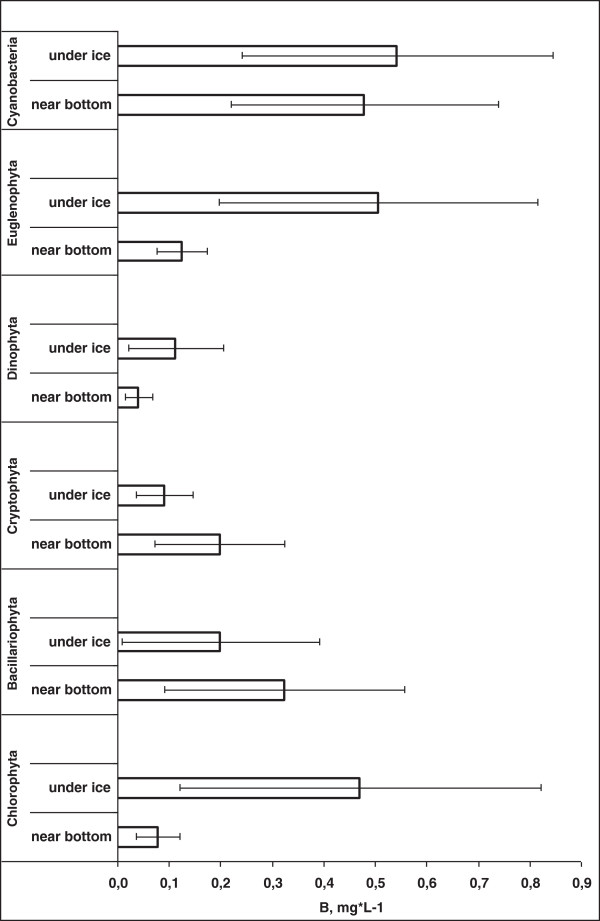
Vertical distribution of the high taxa biomass as the average of the data 1999–2004, 2006 and 2009 years.

Cyanophyta and the flagellates (Euglenophyta and Cryptophyta) were found to be the main components of the under-ice community. Diatoms, Dinophyta or green algae dominated or subdominated the communities only rarely (Figure 3B). The levels of Сyanophyta biomass varied between 0.005 and 3.5 mg L^-1^. As a rule *Limnothrix redekei* was dominant in the samples of high cyanophyta biomass. *Pseudanabaena limnetica*, *Pseudanabaena acicularis*, *Planktolyngbya limnetica* were less often abundant in these samples. Usually, the Planktotrichetum organisms were evenly distributed across the water column (Figure 4).

Euglenophyta were less abundant than Cyanophyta. Its biomass varied at 0.002 - 0.5 mg L^-1^, with the exception of March 2008 when their absolute biomass was between 3.6 - 21.3 mg L^-1^ with *Euglena limnophila* var. *swirenkoi* domination. The vertical distribution of euglenoids varied from year to year: for example, in 1999, the maximum recorded proportion of near-bottom biomass was 91.6%; in 2004, their abundance in the upper layer was 5 times higher than near the bottom.

The next most common algal group was Cryptophyta. Typical biomasses were between 0.001 - 0.86 mg L^-1^. In March 2008, together with the Euglenophyta, the proliferation of this group was exceptionally high under the ice: from 8.6 to 13.4 mg L^-1^, with dominant *Cryptomonas rostrata* Troitzkaja emend. I. Kis. Their vertical distribution peaked in the near-bottom area (Figure 4).

Diatoms biomass of phytoplankton varied between 0.001 - 1.89 mg L^-1^. The major diatom, especially prominent in the near-bottom area, was the eurythermal species *Aulacoseira ambigua*, *Stephanodiscus sp*. and *Fragilaria construens* var. *subsalina* Hust. Generally, the species composition of the diatoms sampled from under the ice was similar to those composing the annual spring blooms. Their contribution to the near-bottom biomass was especially high (Figure 4).

Dinophyta were subdominant only in 2002 and 2007 (Figure 3B). Their biomass varied between 0.01 to 0.57 mg L^-1^. The most common species were *Peridinium aciculiferum* Lemm. and *Peridinium umbonatum* Stein with variations (Additional file 1). The dinophytes were infrequent in near-bottom area (Figure 4).

Green algae were present in trace amounts in all under the ice samples. Biomass of Chlorophyta rarely exceeded 0.02 - 0.5 mg L^-1^. The most common of these algae belonged to genera *Monoraphidium*, *Scenedesmus*, *Pediastrum*, *Chlamydomonas* and *Chlorogonium* (Additional file 1). The green algae were dominant in near-surface samples only in 2004, with domination of *Schroederia setigera* (Shroed.) Lemm., *Chlamydomonas sp*. and *Pteromonas variabilis* Hub. – Pest. Green algae mostly occurred close to the surface (Figure 4).

Chrysophyta comprise small part of total biomass. *Chrysococcus biporus* Skuja, *Chrysococcus punctiformis* Pascher, *Kephyrion rubri*–*claustri* Conrad, *Synura* sp., *Dinobryon* sр. were found to be the most common species.

Coming back to the analysis of non-parameteric correlations (Table 2), we may note the significant relationships between many biotic parameters. Concentrations of total and soluble reactive phosphorus negatively correlated with the relative abundance of Cyanophyta and positively correlated with the relative abundance of the flagellates. The lake’s water level positively but weakly impacted the relative concentration of Cyanophyta and weakly negatively influenced the relative abundance of flagellates. Relative abundance of Cyanophyta strong negative correlated with relative abundance of flagellates (Table 2).

**Figure 5:**
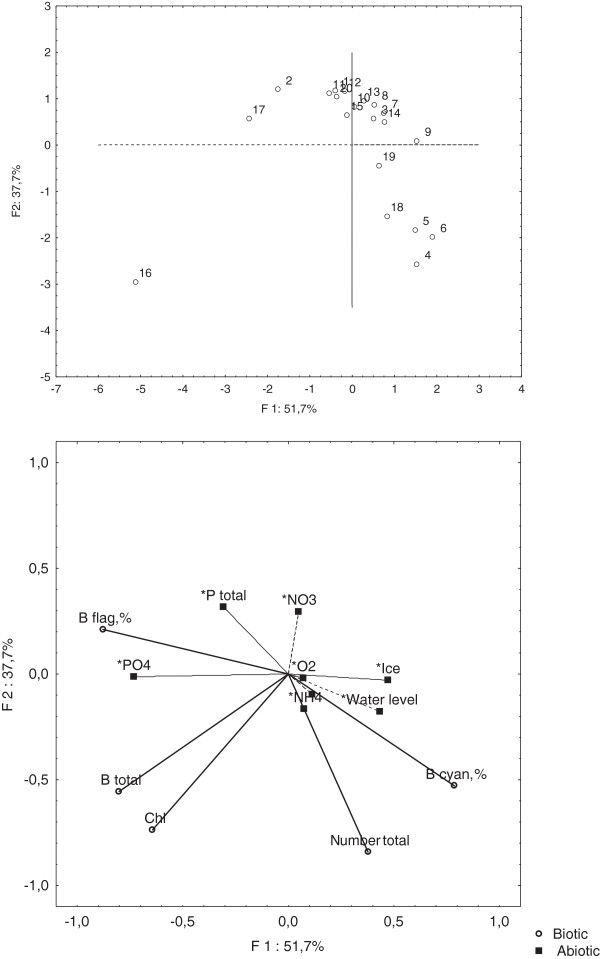
**Plots of the two principal components (PCA) resulting from biotic and abiotic parameters for all samples.** Above - PCA sample scores labeled by number of the sample. Below PCA loading. Abiotic parameters*: NH_4_ – ammonium nitrogen; NO_3_ – nitrate nitrogen; P total – Total phosphorus; Ice – the thick of the ice; O_2_ – oxygen concentrations; Water level – the level of the water. Biotic parameters: B cyan.% - the percent Cyanophyta from total biomass; B flag.% - the percent Euglenophyta and Cryptophyta flagellates from total biomass; Chl – a – chlorophill *a* concentrations, B total – Total Biomass.

The principal-components analyses (PCA) of biotic parameters (relative abundances of Cyanophyta and the Euglenophyta and Cryptophyta flagellates, total number, biomass and concentration of chlorophyll) and abiotic parameters (N-NH_4_, N-NO_3_, P-PO_4,_ P total, O_2_, Ice, Depth) revealed clearly defined groups of samples (Figure 5A). The first and second principal components accounted for 89.5% of total variability. The first component strongly correlated the relative abundances of Cyanophyta, Ice, and Depth and negatively correlated the relative abundances of the flagellates, total biomass, concentration of chlorophyll as well as on the phosphate phosphorus. The samples 2, 16 and 17 comprise a separate cluster according to the first variable. They are distinguished by high total biomass and percent of flagellates exceeding 95% (Figure 5B). The second component negatively correlated to the total number and concentration of chlorophyll and slightly positively depended on nitrate nitrogen and total phosphorus. Concentrations of ammonium and oxygen did not introduce much variation into the first two principal components. The samples 4, 5, 6, 18 and 19, at the lower right corner of the diagram, showed average values of the biomass and percentages of Cyanophyta presence greater than 65%. The compact cloud of observations includes those with low winter biomass values. The dominance of flagellates is depended on the phosphate concentration and Cyanophyta dominate at a higher water level and under thicker ice cover.

## Discussion

Winter is generally considered to be an unfavorable period for phytoplankton growth in boreal, high-latitude regions [3,7,19]. This is often attributed to severe abiotic conditions associated with extended ice cover [15,27,28]. Underwater light conditions in our investigation were not quantified. The observations of Wright [15] suggest that in case of 40 cm thick ice and 22–23 cm of snow only 2% of incident light penetrates this barrier. Our observations include only one period (early 2002) when ice was thin and not covered by snow; even then, the sponge-like structure of the ice restricted its transparency. In other years, ice thickness was around 50 cm, with snow on top to a depth of more than 25 cm; light penetration to and into the water would have been very low at these times. Oxidative demand of the abundant organic matter in the sediments further drives the aqueous oxygen content to anoxia in quite half the samples we collected under-ice. Thus, the conditions towards the end of the period of ice cover in Lake Nero are typified by an almost complete absolute lack of light and oxygen but an abundance in nutrients, especially ammonium nitrogen, phosphate and total phosphorus. The mass mortalities of fish in these lakes in cold winters conform to these circumstances. In spite of the detectable increase of average winter temperature, stabile ice coverage overlaid by the end of winter with the thick layer of snow makes Lake Nero similar to the Scandinavian lakes and distinguishes it from the lakes of Central and South Europe.

While having the harsh abiotic conditions, phytoplankton increase was detected under the ice in bloom proportions in some years (Table 3, Figures 2 and 3). The first, fragmentary data on algal growth under ice in Lake Nero had been obtained in 1988 when low vegetation was detected in March by Lyashenko [29]. Since systematic observations were commenced in 1999, the range of winter phytoplankton abundance has been found to be significant [20,30,31]. In the present paper, we report very high levels of winter algal bloom. A full picture of annual algal succession in the lake is obtained only if appropriate winter observations are included.

Although Lake Nero is hypertrophic, it still harbours a high taxonomic diversity of algae. In observations made between and 1987 – 2008, a total of 800 subgeneric taxa has been recorded [21]. Of these, 172 have been encountered in samples collected when ice covered the lake. This compares favorably with the proportions detected in other eutrophic lakes, situated in similar conditions [3,7,10]. All samples contained *Limnothrix redekei*, usually accompanied by *Pseudanabaena limnetica* and *Planktothrix agardhii*. *Euglena acus*, *Cryptomonas ovata* and *Aulacoseira ambigua* were also found to occur frequently in winter samples. In general, the species composition under the ice of Lake Nero is similar to one found there during the open water period. Although there is a well defined species nucleus under the ice, the Sorensen coefficient between the years was low, thus the species compositions differed considerably from winter to winter.

No compelling trend in the year-to-year changes in under-ice phytoplankton in Lake Nero has been detected. These data are in the same range as that of corresponding data obtained by others for the lakes supporting *Planktotrichetum*-S1 codon (Table 4). Average cell’s volume in winter was higher than in summer (48–13 m^3^ by Babanazarova [30]), although during several winters when the lake was dominated by the S1 species, the average volume was only 20 m^3^. Species with larger cells seem to have advantage in winter phytoplankton. Vertical distribution was heterogeneous and specific for the different phytoplankton groups. Under ice, the plankton was found to be populated by Euglenas, Dinophytes and Chlorophyta. The near-bottom zone was relatively enriched by cryptophytes, some times by euglenoids and diatoms. This is possibly explained by proximity to the sapropel silt, enriched by organic substances, higher temperature in this area and the abundance of the resting stages of algae. During the periods of mass proliferation of the S1 codon the phytoplankton distribution was more even. Examples of similar patterns had been observed by many authors: highly eutrophicated Lake Dautkul in Central Asia [32], the Rybinsk water reservoir [35,36] and small water bodies in North-Western Russia [17].

**Table 4:** **Reported under-ice phytoplankton biomass (mg L**^**-1**^**) in some *Planktotrichetum *S1 codon rich Lakes**

**Lake**	**Research**	**Phytoplankton**	**Reference**
	**period**	**biomass**	
**Petersdorfer See**	1993-1994	5-20	Wiedner & Nixdorf [4]
**Melangsee**	1993-1994	5-10	Wiedner & Nixdorf [4]
**Langer See**	1993-1994	5-20	Wiedner & Nixdorf [4]
**Muggelsee**	1979-1990	1-above 40	Nixdorf & Hoeg [32]
**Lough Neagh**	1973-1974	5-above 40	Jones [33]
**Grizhanku**	1983	5-10	Trifonova [19]
**Labordzhu**	1982-1983	about 1	Trifonova [19]

The abandance of the *Planktothrix* - like cyanobacteria under the ice may depend on the adaptive abilities to very low light incomes and the tolerances to low temperatures [37]. In Lake Grizhanku Trifonova [19] detected high amount of *Planktothrix agardhii* through all winter. In Lake Wolderwijd (Holland), *Planktothrix agardhii*, *Limnothrix redekei* and *Pseudanabaena limnetica* are vegetative almost all year round [38]. Some authors argue that cold winters may interrupt the vegetative cycle of cyanobacteria, while warm winters may benefit them by allowing them to build larger initiating populations, prior to the vernal growth phase [5,39]. Wiedner and Nixdorf [4] investigating lakes Petersdorfer See and Langer See (Germany), put forward the hypothesis that the inverse water stratification and stable, near-bottom temperature of 4°C under-ice conditions provides a temperature refugium for vegetative cyanobacteria, escaping convective mixing nearer the surface entraining colder and near-freezing waters [4]. The prevalence of a *Planktothrix* - like cyanobacteria is typical for Lake Nero, even during the coldest winters, that could be supported the hypothesis of C. Wiedner and B. Nixdorf. Coexistence of low amounts of the S1 codon species through all winters including very low levels biomass confirms the importance of the functional group concept in phytoplankton ecology.

High proliferation of flagellates belonging to the Euglenophyta and Cryptophyta and the high frequencies in samples, may be regarded as a peculiarity of the under-ice period for Lake Nero too and allows one to treat them from the point of view of functional groups [20]. The preponderance of Euglenoids at meso-eutrophic ponds and shallow lakes belonging to W2 codon by phytosociological nomenclature [23]. Large cryptomonads and small dinoflagellates refers to a wide range of habitats, which reflect the ability of its representative in conditions when grazing pressure is low belonging to codon Y [23,40]. Codons W2 and Y played a significant role in the community of Lake Nero only in winter. A similar circumstance has been described for hypertrophic Lake Utah (USA), where concentrations of mineral and organic phosphorus are very high [3], and in shallow lakes of North Dakota, USA [7]. The euglenoids and cryptomonads flagellates are well known to typify cold waters, tending to prosper under ice in water-bodies of various types, invoking their cold-resistance, mobility and mixotrophy [7,15,19,28,41,42]. Almost complete disappearance of zooplankton towards the end of winter is peculiar to Lake Nero [26]. This also favors net development of W2-Y flagellates as low grazing pressure conditions. In our studies the principal component analysis suggests that the W2-Y flagellates respond to high concentrations of phosphorus. The lesser role of dinoflagellates in Lake Nero may be explained by the almost complete lack of oxygen needed by these algae [43].

In Lake Nero, the fraction of flagellates (W2-Y codon) was a small part of the biomass in the years when S1 algae were blooming. This observation is supported statistically. Therefore, the composition of the winter phytoplankton in Lake Nero, involves either the proliferation of species of S1 *Planktorhrix* – like cyanobacteria or of the species of W2-Y flagellates. The statistical analysis suggests that, during the coldest winters under thick ice and anoxic conditions, and in the presence of high ammonium nitrogen, S1 enjoy a considerable functional and selective advantage. Conversely, warmer winters and higher phosphorus concentrations are more favorable to the W2-Y euglenoids and cryptomonads flagellates.

## Conclusion

This study provides evidence that conditions of near anoxia, of severe light deficiency and high concentration of biogenic elements in the end of winter in the hypertrophic lake present highly selective conditions for proliferation of most phytoplankton. The abundance of the phytoplankton varies from trace amounts to an under-ice bloom level. A full picture of algal succession in the lake may be obtained only if systematic winter observations are taken into account. The dominant role of *Planktothrix*-like cyanobacteria (S1 codon) which is observed from early summer to late autumn may persist through winter and even a small amount of winter survivors belonging to the summer dominants are sufficient for their reestablishment in summer. However here we show that the switch of the dominant organism to the euglenoids and cryptomonads (W2-Y codons) is likely to occur.

## Methods

### Description of the locality studied

Lake Nero is situated in the Upper Volga River Basin. It is the largest lake in Yaroslavl province; its area is about 58 km^2^; its maximum depth is 4.7 m; mean depth is 1.6 m. The lake was formed in the Late Pleistocene period, ca. 150 000 years ago, during the wasting of the Moscow Glacier. During the last 5000 years, starting at the end of the Boreal period or the beginning of the Atlantic Period, the lake has been shallow, inhabited by species typical of eutrophic water bodies. Lake Nero has 12 inflowing rivers, the largest of which is Sara River. There is one outlet to the lake: Veksa Rostovskaya River, which flows to the Kotorosl’ River and which is, in turn, a tributary of the Volga River. The water level has been regulated by the dam from the end of 1980. The area of the watershed is 1314 km^2^; the water exchange rate is 1,9 times per year. The chemical composition of the lake differs from that of other lakes of the area: it contains more minerals , with a high concentration of chloride [25]. The bottom of the lake is covered by a thick (average thickness 4.9 m) layer of sapropelic silt, which accumulates biogenic elements. The northern part of the lake is open and is dominated by phytoplankton; the southern part supports macrophytes on 15% emergent and 9% of submerged "swamp" [44].

### Sampling and calculations

**Figure 6:**
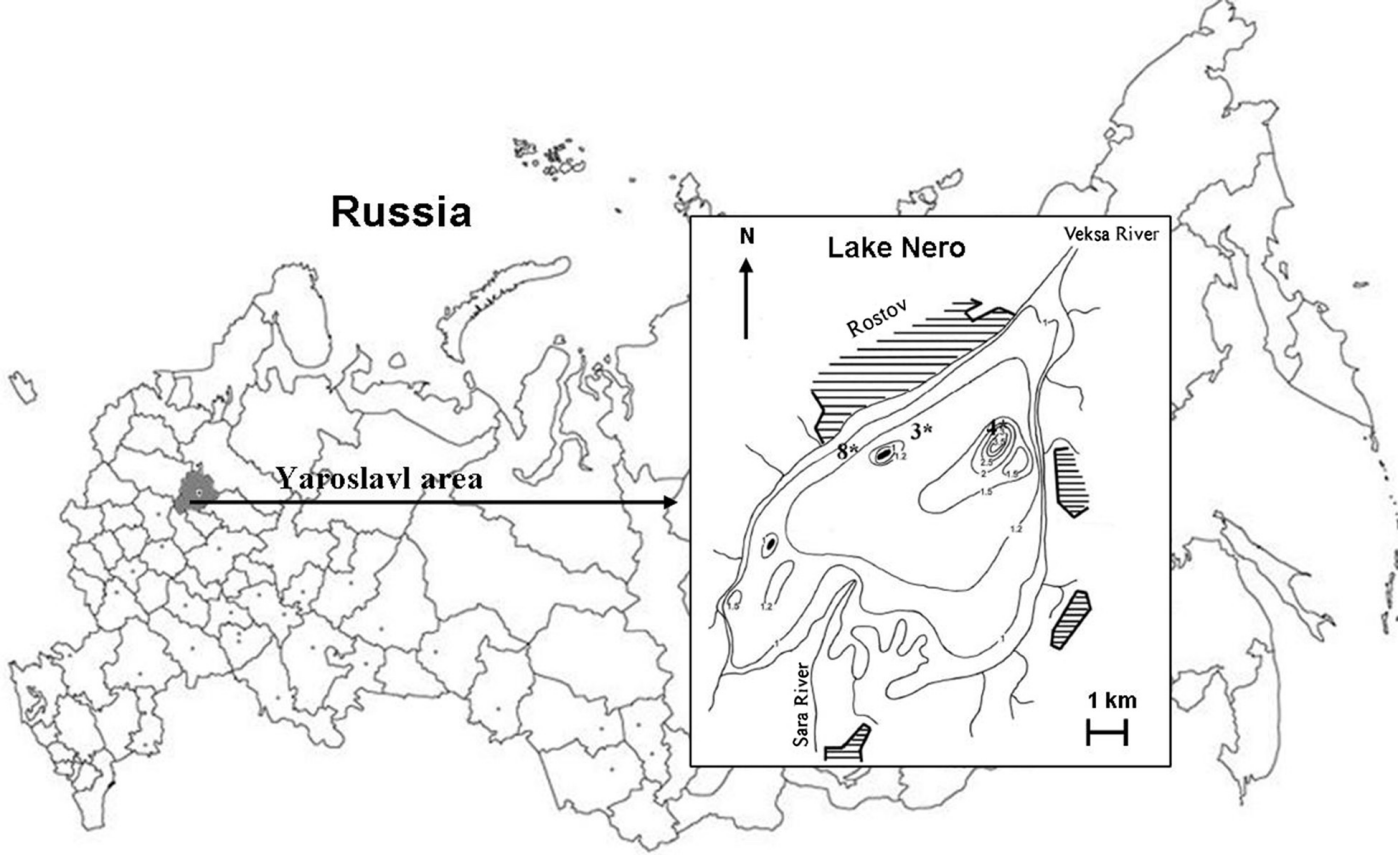
Lake Nero sampling station (3*,8*,4*); insert: geographical location in Russia.

The samples were collected under-ice in March of 1999, 2002–2010 from one to three stations, situated in Northern and Central parts of the lake, close to the city of Rostov (Figure 6). Contrasting weather conditions were observed. The winters of 1999, 2004 and 2008 were relatively mild. However, those of 2003, 2005 and 2010 were extremely cold. Samples were collected by Ruttner bathometer from just beneath the ice surface and near the bottom of the lake in 1999–2004, 2006 and 2009, at other years only under ice by weathers circumstances. The identification and counting of phytoplankton, analysis of pigments (chlorophyll *a*) and biogenic elements were done from one bathometer. Samples for chemical analysis were fixed in the field with chloroform and processed on return to the laboratory. Soluble phosphorus was determined colorimetrically using the sulfate / molybdate method; ammonium, nitrate and nitrite nitrogen were determined using Nessler reagent, sulfaphenol and Griss chemical [45]. Photosynthetic pigments were extracted from filter residues, using acetone, and measured spectrophotometrically; concentrations of chlorophyll *a* and phaeopigments were calculated according to the method and equations of Lorenzen [46]. Phytoplankton was fixed with Lugol’s Iodine and enumerated in a Najötta chamber according to the Utermohl method. Tabulated information included the taxonomic identity of the organisms (as far as possible), the number and sizes of cells. Individual cell volumes were calculated by approximation of cell shapes to simple geometric figures. Aggregate algal biomass was determined as the sum of the products of species-specific counts and individual volumes. Species comprising more than 10% of total biomass were considered to be dominant. The ecological characteristics of phytoplankton is based on lists of indicator species saprobity, salinity and pH [47-49]. Shannon-Weaver index [50] was used for analyses of phytoplankton diversity. For the comparative analysis of phytoplankton between different years, the Sorensen index (K) was used:

$$K={2N}_{a+b}/N_{a}+N_{b},$$

where N_a+b_ is the number of species in common detected in the samples a and b, and N_a_ and N_b_ is the number of species in the samples a and b.

This study is based on analysis of 32 samples and sets of limnological observations. Coefficients of correlation and factor analyses were solved using conventional methods as adopted in the STATISTICA software, version 6.0 (StatSoft, Inc.) and MS Excel programs.

## Competing interests

The author’s declare that they have no competing interests.

## Authors’ contributions

BO did the field work, the identification and counting of phytoplankton, performed the statistical analysis and wrote the manuscript. SS made the analysis of pigments (chlorophyll *a*) and of elements of biolological importance and co-wrote the manuscript. SSch was involved in the flora characterization as well in the description of the habitat and co-wrote the manuscript. All authors have read and approved the final manuscript.

## Authors’ information

Yaroslavl State University named by P.G. Demidov, proezd Matrosova 9, Yarosalvl, Russia, Department Ecology and Zoology

## Supplementary Material

Additional file 1 Taxonomic composition and eco-geographical characteristics of the under-ice phytoplankton in the lake Nero (1999-2010). Click here for file
