# Supplementary material for: The structure of winter phytoplankton in Lake Nero, Russia, a hypertrophic lake dominated by Planktothrix-like Cyanobacteria
Source: Aquat Biosyst. 2013 Sep 30;9:18. doi: 10.1186/2046-9063-9-18 (PMC3849563; doi:10.1186/2046-9063-9-18)
Supplement: Additional file 1 — Taxonomic composition and eco-geographical characteristics of the under-ice phytoplankton in the lake Nero (1999-2010). [file 2046-9063-9-18-S1.pdf]

PRIMARY RESEARCH PAPER

Olga Babanazarova, Sergey Sidelev, Svetlana Schischeleva

The structure of winter phytoplankton in Lake Nero, Russia, a hypertrophic lake dominated by *Planktothrix*-like Cyanobacteria

Taxonomic composition and eco-geographical characteristics of the under-ice phytoplankton in the lake Nero (1999-2010)

Taxon	Habitat	Distribution	Salinity	pH	Saprobity
**Cyanophyta (Cyanoprokaryota)**					
**Chroococcales**					
Aphanocapsa delicatissima W. et G.S. West.	P	c	–	–	–
A. grevillei (Berk.) Rabenh.	L	c	–	Al	o–β
Gloeocapsa sp.	–	–	–	–	–
Microcystis aeruginosa (Kütz.) Kütz.	P	c	–	Al	β
M. flos-aquae (Wittr.) Kirchner	P	c	IN	–	–
Synechocystis sp.	–	–	–	–	–
**Nostocales**					
Anabaena cylindrica Lemm.	P	–	–	–	o–β
Anabaena sp.	–	–	–	–	–
Aphanizomenon gracile Lemm.	P	c	H	–	–
**Oscillatoriales**					
Geitlerinema amphibium (Ag. ex Gom.) Anagn.	L	c	–	–	–
Jaaginema geminatum (Menegh. ex Gom.) Anagn. et Kom.	P	c	–	In	–
Limnothrix planctonica (Wolosz.) Meffert	P	c	–	–	o–β
L. redekei (Van Goor) Meffert	P	c	IN	–	β
Oscillatoria sp.	–	–	–	–	–
Phormidium amoenum Kütz. ex Anagn. et Kom.	O	–	IN	–	–
P. granulatum (Gardner) Anagn.	P	b	IN	–	–
P. molle Gom.	L	c	IN	–	β

Phormidium sp.	–	–	–	–	–
Planktolyngbya circumcreta (G.S. West) Anagn. et Kom.	P	–	IN	–	–
P. contorta (Lemm.) Anagn. et Kom.	P	–	IN	–	–
P. limnetica (Lemm.) Kom.-Legn. et Cronb.	P	c	–	In	β–α
Planktolyngbya sp.	–	–	–	–	–
Planktothrix agardhii (Gom.) Anagn. et Kom.	P	c	IN	–	β
Pseudanabaena acicularis (Nyg.) Anagn. et Kom.	P	–	IN	–	–
P. limnetica (Lemm.) Kom.	P–B	c	IN	–	o–β
P. mucicola (Naum. et Hub.–Pestal.) Schwabe	P	c	IN	–	o–β
Romeria leopoliensis (Racib.) Koczw.	P	–	–	–	o–β
**Chrysophyta**					
**<u>Chromulinales</u>**					
Chromulina Cienk. *sp.*	–	–	–	–	–
Chrysococcus biporus Skuja	P	c	IN	In	o–β
C. punctiformis Pascher	P	–	IN	In	o–β
C. triporus Matv.	P	–	IN	In	o–β
Kephyrion moniliferum (Schmid.) Bourelly	–	–	–	–	–
K. rubri–claustri Conrad	L	b	IN	–	o
K. spirale (Lackey) Conrad	L	–	IN	–	β
Kephyrion sp.	–	–	–	–	–
**<u>Ochromonadales</u>**					
Dinobryon sp.	–	–	–	–	–
Mallomonas sp.	–	–	–	–	–
Microglena punctifera (Müller) Ehr.	L	c	IN	–	–
Ochromonas sp.	–	–	–	–	–
Pseudokephyrion conicum (Schiller) Schmid	–	–	–	–	–
Synura sp.	–	–	–	–	–
**Bacillariophyta**					
**<u>Thalassiosirales</u>**					
Cyclostephanos invisitatus (Hohn et Hell.) Ther., Stoer. et Håk.	P	c	IN	Al	–
Cyclotella sp.	–	–	–	–	–
Stephanodiscus binderanus (Klitz.) Krieg.	–	–	–	–	–
S. hantzschii Grun.	P	c	IN	Al	α-p

S. minutulus (Kütz.) Cleve et Möller	P	b	IN	Al	α
Stephanodiscus sp.	–	–	–	–	–
**Melosirales**					
Aulacoseira ambigua (Grun.) Sim.	P	c	IN	Al	o–β
A. granulata (Ehr.) Sim.	P	c	IN	Al	β
**Araphales**					
Fragilaria capucina var. *radians* (Kütz.) Lange-Bert.	P	c	IN	Al	o–β
Staurosira construens var. *construens* Ehr.	L	c	IN	Al	o–β
S. construens var. *binodis* (Ehr.) Ham.	O	c	IN	Al	o
S. construens var. *exigua* (Smith) Kobayasi	L	c	IN	Al	–
Staurosirella berolinensis (Lemm.) Bukht.	P	c	IN	Al	β
Synedra sp.	–	–	–	–	–
Ulnaria acus (Kütz.) Aboal	P	c	IN	Al	o–β
U. delicatissima var. *angustissima* (Smith) Aboal et Silva	P	c	IN	Al	o–β
**Raphales**					
Cymatopleura solea (Breb.) Smith	L	c	IN	Al	β
Gyrosigma acuminatum (Kütz.) Rabenh.	O	b	IN	Al	β
Navicula sp.	–	–	–	–	–
Nitzschia acicularis (Kütz.) W. Sm.	P	b	IN	Al	α
N. gracilis Hantzsh.	P	c	IN	In	β
N. holsatica Hust.	P–B	c	IN	In	–
N. longissima (Bréb.) Ralfs	–	–	–	–	–
N. palea var. *debilis* (Kütz.) Grun.	P–B	c	H	In	o
N. paleacea Grun.	P–B	c	IN	Al	β–α
N. recta Hantzsch	L	c	–	Al	β–α
N. sublinearis Hust.	B	b	IN	–	o–β
N. vermicularis (Kütz.) Hantzsch	L	c	IN	Al	β
Pinnularia major (Kütz.) Rabenh.	B	c	IN	In	o
P. microstauron (Ehr.) Cleve	B	c	–	In	–
Surirella biseriata Bréb	P	c	IN	Al	β
**Xanthophyta**					
**Heterococcales**					
Tetraedriella impressa Pasch.	P	–	–	–	–

**Cryptophyta**					
**<u>Cryptomonadales</u>**					
Chroomonas minima Czosnow.	–	–	–	–	–
C. caudate Geitler	P	c	IN	–	β–α
Cryptomonas caudata Schiller	P	c	IN	–	–
C. curvata Ehr.	P	c	–	In	β
C. erosa Ehr.	L	c	H	In	α
C. obovoidea Pascher	P	–	–	–	–
C. marssonii Skuja	P	c	IN	–	o–β
C. nasuta Pasch.	L	–	H	–	–
C. ovata Ehr.	P	c	IN	In	α
C. parapyrenoidifera Skuja	–	–	–	–	–
Cryptomonas sp.	–	–	–	–	–
**Dinophyta**					
**<u>Peridiniales</u>**					
Gymnodinium sp.	–	–	–	–	–
Peridiniopsis sphaeroideum (Christen) Bourrelly	P	–	–	–	–
Peridinium aciculiferum Lemm.	P	c	–	In	o–β
P. umbonatum var. *umbonatum* Stein	P	c	IN	In	o–β
P. umbonatum var. *deflandrei* (Lefevre) Pop. and Pfiester	P	–	–	–	–
P.umbonatum var. *goslaviense* (Wolosz.) Pop. and Pfiester	P	–	–	–	–
P. umbonatum var. *incospicuum* Stein.	–	–	–	–	–
Peridinium sp.	–	–	–	–	–
Woloszynskia tenuissima (Lauterb.) Thomp.	–	–	–	–	–
**Euglenophyta**					
**<u>Euglenales</u>**					
Euglena acus Ehr.	L	c	IN	In	β
E. acus var. *minor* Hansg.	–	–	–	–	–
E. gracilis Klebs	L	c	IN	Al	o–β
E. granulata (Klebs) Schmitz	L	c	IN	–	–
E. hemihromata Skuja	L	–	–	–	β
E. limnophila Lemm.	L	–	–	–	o–β
E. limnophila var. *swirenkoi* (Arnoldi) Pop.	L	–	IN	–	–

E. megalitus Skuja	P	–	–	–	–
E. minima France	L	c	–	–	o
E. pisciformis Klebs	L	c	IN	–	β–α
E. proxima Dang.	P	b	IN	In	o–β
E. vagans Delf.	P–B	c	–	–	–
E. viridis Ehr.	P–B	c	–	In	α-p
Euglena sp.	–	–	–	–	–
Phacus arnoldii var. *ovatus* Popova	–	–	–	–	–
P. indicus Skvortzov	P	c	IN	In	o–β
P. oscillans Klebs	L	c	H	–	–
P. parvulus Klebs	L	c	IN	In	β
P. pyrum (Ehr.) Sein	P	c	IN	In	o–β
P. striatus France	L	–	–	–	–
Phacus sp.	–	–	–	–	–
Strombomonas acuminata (Schmarda) Defl.	L	c	IN	In	o–β
Trachelomonas hispida (Perty) Stein emend. Defl.	P	c	IN	In	β
T. hispida var. *crenulatocollis* (Maskell) Lemm.	P	c	IN	In	–
T. hispida var. *granulata* Playf.	L	c	IN	In	–
T. hispida f. *punctata* Lemm.	–	–	–	–	–
T. hispida var. volicensis Drez.	P–B	–	–	–	–
T. intermedia Dang.	P	c	IN	In	o
T. planctonica var. *oblonga* Drez.	P	c	IN	–	–
T. rotunda Swir.	L	c	IN	In	o
T. spinulosa (Skv.) Defl.	P	c	IN	Al	–
T. volvocina Ehr.	P	c	IN	In	o–β
T. volvocina var. *punctata* Playf.	P	c	IN	–	–
T. volvocina var. *subglobosa* Lemm.	P	c	IN	In	o–β
Trachelomonas sp.	–	–	–	–	–
**Chlorophyta**					
**<u>Chlorellales</u>**					
Closteriopsis acicularis (G.M. Sm.) Belcher et Swale	P	c	IN	Al	–
Koliella longiseta (Visch.) Hind.	L	c	IN	In	β
K. planctonica Hind.	P	–	–	–	–

**Chlorococcales**					
Chlorococcum sp.	–	–	–	–	–
Golenkinia radiata Chod.	P	c	IN	–	β
**Chlorodendrales**					
Tetraselmis cordiformis (Carter) Stein	P	–	–	–	–
**Oocystales**					
Lagerheimia genevensis (Chod.) Chod.	P	c	IN	–	β
**Sphaeropleales**					
Ankistrodesmus falcatus (Corda) Ralfs	L	c	IN	–	β
A. gracilis (Reinsch) Korsch.	P	c	IN	Al	β
Coelastrum astroideum De Not.	P	c	IN	In	β
C. microporum Näg.	P	c	IN	–	β
Hyaloraphidium contortum Pasch. et Korsch.	L	c	IN	–	–
Kirchneriella contorta (Schmidle) Bohl.	P	c	IN	–	–
K. obtusa (Korsch.) Kom.	P	c	IN	–	–
Monoraphidium arcuatum (Korsch.) Hind.	P	c	IN	–	β
M. contortum (Thur.) Kom.–Legn.	P	c	IN	–	β
M. irregulare (Smith) Kom.–Legn.	P	c	IN	In	–
M. komarkovae Nyg.	P	c	IN	In	–
M. minutum (Näg.) Kom.–Legn.	P	c	IN	Al	β–α
Pediastrum biradiatum Meyen	P	c	IN	In	β
P. boryanum (Turp.) Menegh.	P	c	–	In	β
P. boryanum var. *longicorne* Reinsch	P	c	IN	In	–
P. duplex Meyen	P	c	IN	–	β
P. simplex Meyen var. *simplex*	P	c	IN	–	o–β
P. simplex var. *echinulatum* Wittr.	P	c	IN	–	–
P. tetras (Ehrenb.) Ralfs	P	c	IN	–	β
Scenedesmus acuminatus (Lagerh.) Chod.	P	c	IN	–	β
S. communis Hegew.	P	c	–	–	–
S. ecornis (Ehr.) Chod.	P	c	–	–	β
S. quadricauda (Turp.) Breb.	L	c	–	In	β
S. spinosus Chod.	P	c	IN	–	–
Scenedesmus sp.	–	–	–	–	–

Schroederia setigera (Shroed.) Lemm.	P	c	IN	–	β
Tetraedron caudatum (Corda) Hansg.	P	c	IN	–	β
T. minimum (Braun) Hansg.	P	c	IN	–	β
**<u>Volvocales</u>**					
Chlamydomonas actinochloris Deason et Bold	–	–	–	–	–
C. augustae Skuja	–	–	–	–	–
C. kvildensis Ettl	P	–	H	In	–
Chlamydomonas sp.	–	–	–	–	–
Chlorogonium gracile Matw.	P	–	–	–	–
C. maximum Skuja	–	–	–	–	–
Pteromonas variabilis Hub. – Pest.	–	–	–	–	–
Sphaerellopsis cylindrica Skuja	P	–	IN	–	–
**<u>Zygnematales</u>**					
Staurastrum tetracerum Ralfs var. *tetracerum*	P	–	–	–	o–β

1. Habitat: P – planktonic, L – littoral, B – benthic. 2. Distribution: c – cosmopolitan, b – boreal. 3. Salinity: IN – indifferent , H – halophilous. 4. pH: Al – alkaliphilous, In – indifferent. 5. Saprobity: o – oligosaprobous, β – β-mesosaprobous, α – α-mesosaprobous, p – polysaprobous.
